# Building a family network from genetic testing

**DOI:** 10.1002/mgg3.259

**Published:** 2016-12-29

**Authors:** Kathleen A. Leppig, Heidi A. Thiese, David Carrel, David R. Crosslin, Michael O. Dorschner, Adam S. Gordon, Andrea Hartzler, James Ralston, Aaron Scrol, Eric B. Larson, Gail P. Jarvik

**Affiliations:** ^1^ Genetic ServicesGroup Health CooperativeSeattleWA98112USA; ^2^ Department of PathologyUniversity of WashingtonSeattleWA98195USA; ^3^ Group Health Research InstituteGroup Health CooperativeSeattleWA98101USA; ^4^ Department of Biomedical Informatics and Medical EducationUniversity of WashingtonSeattleWA98195USA; ^5^ Department of Medicine (Medical Genetics) and Genomic SciencesUniversity of WashingtonSeattleWA98195USA

**Keywords:** Electronic medical record and genomics, family network, *LDLR*, *RYR1*, *SCN5A*

## Abstract

**Background:**

Genetic testing has multigenerational and familial repercussions. However, the “trickle‐down effect” of providing genetic counseling and testing to family members at risk after an initial identification of a pathogenic variant in a medically actionable gene has been poorly understood.

**Methods:**

Three probands were identified during the pharmacogenetics research phase of eMERGEII (electronic MEdical Record and Genomics, phase II) to have variants in genes associated with autosomal dominant adult‐onset disorders determined to be actionable by the American College of Medical Genetics (ACMG). Two of the three probands had variants that were classified as pathogenic and the third proband had a variant ultimately classified of uncertain significance, but of concern due to the proband's own phenotype. All probands had additional family members at risk for inheriting the variant. Two of the three probands had family members who received their medical care from the same health care system, Group Health Cooperative (GHC). It was recommended that the proband contact their family members at risk to be referred to genetic counseling for consideration of genetic testing.

**Results:**

The two probands with pathogenic variants contacted some of their family members at risk. Individuals contacted included children and adult grandchildren, particularly if they received their medical care at GHC. To the best of our knowledge, siblings and more distant relatives at risk were not informed by the proband of their genetic risk.

**Conclusions:**

Establishing a family network is essential to disseminate knowledge of genetic risk. These three initial cases describe our experience of contacting eMERGE participants with identified variants, providing the probands with appropriate genetic counseling and care coordination, and recommendations for contacting family members at risk. Greater challenges were observed for coordinating genetics care for family members and extending the family network to include other relatives at risk.

## Introduction

As more patients have genomic testing for clinical reasons or as part of research programs such as the electronic MEdical Records and GEnomics (eMERGE) Network, Clinical Sequencing and Exploratory Research (CSER), and Precision Medicine Initiative (PMI), more individuals will receive results from their genomic testing that are not related to their reason for enrollment. Many of the results will represent highly penetrant pathogenic variants, some of which are associated with phenotypes that are medically actionable. These will have medical implications for biological family members. We call these results additional findings, due to patient preference for that term over the terms incidental or secondary findings (Tan et al. 2016).

Medical geneticists have been challenged to determine how to disseminate information throughout an at‐risk family about the potential risk for a genetic condition given legal, ethical, and social issues. The “duty to warn” (Offit et al. 2004) has been raised in cancer genetics but can be extended to pathogenic variants in other actionable, non‐cancer genes. However, any such duty can be in direct conflict with patient or participant privacy rights. Ideally when a pathogenic variant is identified in a proband, they are the best person to contact at‐risk family members with information about genetic counseling and options for genetic testing. It has been shown that increased communication and follow‐up testing in families increases the cost‐effectiveness of genetic testing for colorectal cancer risk (Gallego et al. 2015). However, such ideal process for “trickle‐down testing” for extended family members has yet to be defined.

Our pharmacogenomics research project within phase II of the eMERGE (eMERGEII) Network offered a setting through which to examine the “trickle‐down effects” of genetic testing for extended family members. eMERGE is an NHGRI‐supported consortium of biorepositories combined with electronic health records (EHR) for conducting genomic studies that includes a special emphasis on related ethical, legal, and social issues (McCarty et al. 2011) Group Health Cooperative (GHC) and University of Washington (UW) have collaborated as an eMERGE site since phase I (Bush et al. 2016). GHC is an integrated health care system founded in 1945 with nearly 600,000 members; many extended families receive the care through this system.

In eMERGEII, three probands enrolled in the study received additional results in genes considered actionable by the ACMG (Green et al. 2013). In two of the three probands, the variants were pathogenic. We describe our experience with the three probands and their at‐risk family. For those family members who received their health care through GHC, coordination of genetic testing and communication of results were provided by GHC Genetic Services. For these three families, we will delineate the additional impact on the health care system based on the identification of their genetic risk. These cases serve as examples of the importance of building a family network for patients with highly penetrant genetic variants to enable appropriate health care and surveillance for individuals at risk.

## Methods

All three probands were among thousands enrolled to participate in genomic research studies performed collaboratively between GHC and the UW and completed an IRB‐approved consent process (GHC IRB 486548). Two patients were recruited as part of a pharmacogenomics study through eMERGE. The participants specifically agreed to participate in a research study using sequencing to identify pharmacogenomics variants and to return of actionable information (Bush et al. 2016).

The Nickerson laboratory at the UW performed DNA sequencing for 82 pharmacogenes using the PGRNSeq platform (Gordon et al. 2016). These data included sequence results for several genes on the ACMG list of 56 gene‐disease pairs considered actionable (Green et al. 2013) for pathogenic, high penetrance variants, including *SCN5A, RYR1*,* and LDLR*. With approval from Group Health Research Institute IRB, the three patients were contacted by phone regarding their results by either a medical geneticist or internal medicine physician and asked to make an appointment with Genetic Services at GHC to discuss the implications of the findings and arrange appropriate follow‐up and management. All three patients, described below, were evaluated in Genetic Services and in two of the three cases, additional family members at risk were identified, some of whom were receiving health care through GHC.

To contact family members at risk, we requested that the proband inform their family members and have those who receive their medical care through GHC contact Genetic Services to arrange for the appropriate genetic testing. Genetic testing was coordinated for at‐risk family members and results were disclosed by a genetic counselor. For adult patients who tested positive for the familial pathogenic variant, Genetic Services then coordinated follow‐up, management and surveillance. For family members who did not receive their healthcare at GHC, we suggested the proband contact family members by either verbal or written communication. We offered to provide sample letters used to communicate genetic results to family members and information on how to locate a nearby genetic clinics.

### Proband 1

The proband is a 68‐year‐old female who was found to have a pathogenic missense variant in the *SCN5A* gene (OMIM 600163), c.1603G>A, resulting in a truncating mutation p.Arg535X. *SCN5A* is a sodium channel gene with variants associated with Brugada syndrome and other inherited cardiac conduction abnormalities. (Sarquella‐Brugada et al. 2016). Truncating mutations in *SCN5A* have been associated with increased cardiac dimensions, reduced cardiac contractility, and cardiac conduction defects including Brugada syndrome (Niu et al. 2006; van Hoorn et al. 2012). Moreover, truncating mutations have been shown to result in mutant channel expression with subsequent degradation (Ziyadeh‐Isleem et al. 2014). The proband's past medical history was remarkable for hypertension, hyperlipidemia, obesity, and coronary artery disease, including coronary artery stent placement with subsequent myocardial infarction. She reported one episode of syncope but no history of seizures during her lifetime. She has not had an EEG performed. The patient had a history of depression and was being treated with bupropion without any adverse effects. Other medical problems included irritable bowel syndrome and osteopenia.

Clinical studies include an EKG which showed sinus bradycardia with a first‐degree A‐V block and left bundle branch block but without evidence of Brugada syndrome. The echocardiogram showed mildly impaired left ventricular diastolic function and mildly elevated pulmonary artery systolic pressure.

### Family history 1

**Figure 1:**
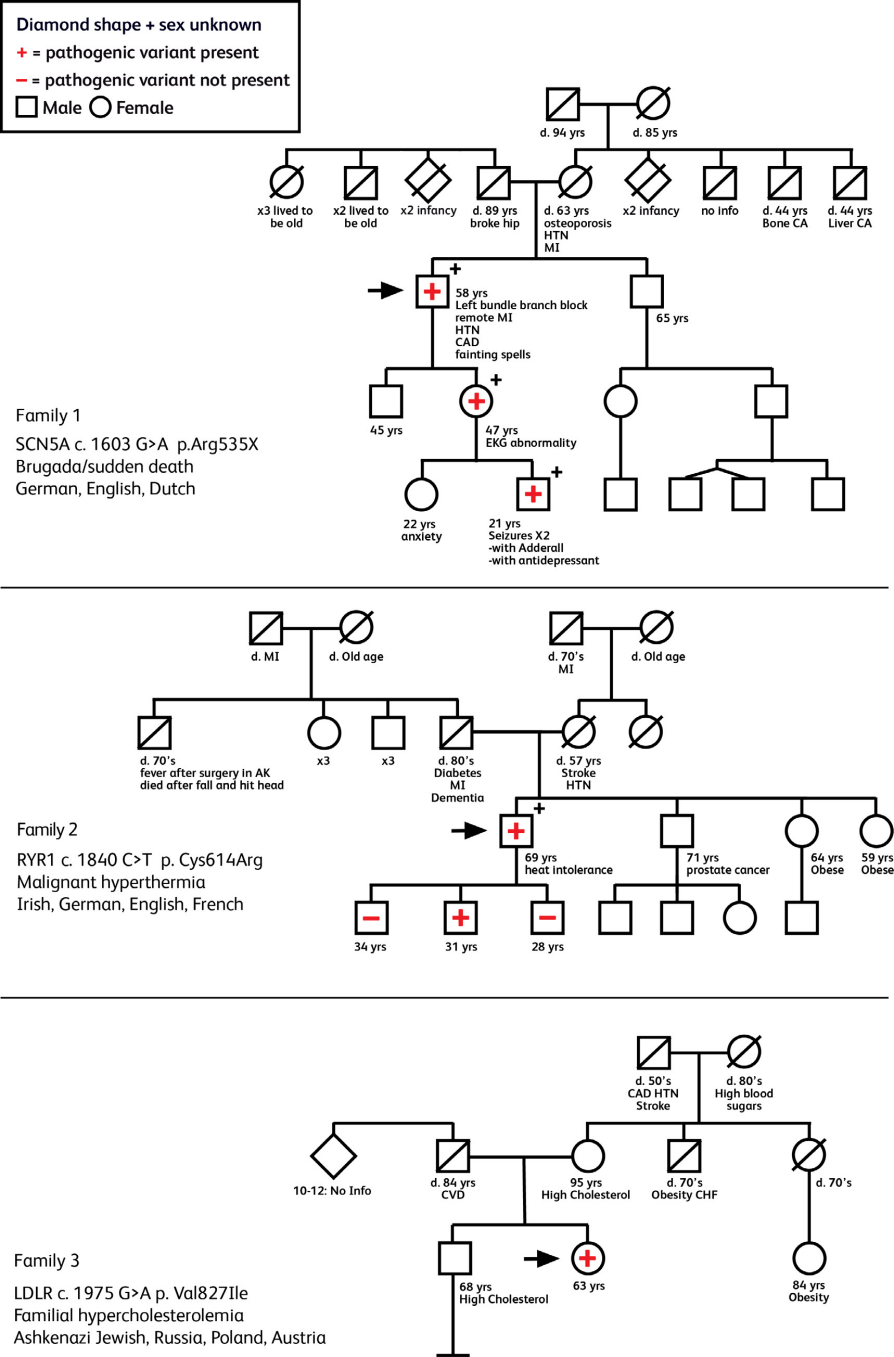
Pedigrees of Families, 1, 2, and 3. Specific gene and pathogenic variant indicated per each family. Arrow indicates proband. “+” indicates testing positive for the familial pathogenic variant while “−” indicates testing negative for the familial pathogenic variant.

The father of the proband died at 89 years of age, had diabetes mellitus type 2 and complications from a hip fracture (Fig. 1). The mother of the proband died of a myocardial infarction at age 63 and had hypertension and osteoporosis. The proband had two children, a 47‐year‐old daughter who received her healthcare at GHC and a 45‐year‐old son who lived outside the state of Washington and reported no heart symptoms. At this time, it is unknown whether the proband's son pursued genetic testing.

The proband's daughter reported a prior history of EKG abnormality identified as an intraventricular conduction defect (IVCD). Additional medical history was remarkable for hypercholesterolemia, obesity, and depression. Genetic testing showed that the proband's daughter shared the *SCN5A* pathogenic variant. A subsequent EKG showed a prolonged QRS, but no evidence of Brugada syndrome. Echocardiogram showed mildly impaired left ventricular diastolic function and mild aortic regurgitation.

This daughter of the proband had adult children in their 20's both of who receive their health care at GHC including a daughter who has not pursued genetic counseling or testing and a son with a history of epilepsy and fainting spells. This 21‐year‐old male was diagnosed with ADHD at 6 years of age and began treatment with dextroamphetamine. He had his first seizure at 7 years of age and was subsequently placed on Dilantin. This patient described recurrent childhood episodes of blanking out or freezing, particularly following physical activity. He had a normal EKG and EEG as a child. The family decided to discontinue all medications at 8 years of age because of concern of side effects. The patient did well until 18 years of age when he was treated for depression with citalopram. Within a month of starting the medication, he fainted while taking a shower. No EEG or EKG was performed when he was evaluated in urgent care on the day of the event. Antidepressant medication was discontinued.

The genetic testing of the proband's grandson was positive for the familial *SCN5A* pathogenic variant. A subsequent EKG showed nonspecific interventricular conduction delay, but no evidence of Brugada syndrome. An echocardiogram showed mildly impaired left ventricular diastolic function, mild pulmonic valve insufficiency, and frequent premature ventricular contractions. A 24‐h Holter monitor showed sinus rhythm with isolated PVC's and without evidence of heart block. Annual follow‐up in cardiology is planned.

### Proband 2

The proband was a 70‐year‐old man found to have a pathogenic variant, c. 1840 C>T, pArg614Cys in the *RYR1* gene (OMIM 180901)that was previously reported to be associated with malignant hyperthermia (Gillard et al. 1991; Gonsalves et al. 2013). The patient had a medical history including diabetes mellitus type 2, hypertension, benign prostatic hypertrophy, and osteoarthritis. The proband had three surgical procedures during his lifetime, but no episodes of malignant hyperthermia or rhabdomyolysis. However, he reported feeling feverish and had difficulty cooling down on multiple occasions after hiking in extreme heat.

### Family history 2

The proband's father had type 2 diabetes and dementia, and died of a myocardial infarction in his 80's (Fig. 1). A paternal uncle developed a high fever in association with a minor surgery requiring anesthesia as a child in a rural Alaskan hospital. He died in his 70's from an accidental head injury. In addition to the proband's father and uncle, there were six other individual in the sib ship, none with a history suggestive of malignant hyperthermia. The proband's mother died of hypertension and a stroke at age 57.

The proband had three siblings, one brother and two sisters. The 71‐year‐old brother has prostate cancer. His brother and one sister have had surgical procedures performed during their lifetime, without complications of hyperthermia. The proband had three sons all of whom reported to be in good health. Two of the three sons received their health care at GHC, were seen in Genetics Clinic for genetic counseling, completed genetic testing and had results disclosure. The third son of the proband lived in Washington State, but received his healthcare outside of GHC. He did not want to be evaluated at another institution that provides genetic services, and requested that his genetic testing by coordinated through his primary care provider. After more than five attempts, his genetic testing was completed. One son, age 31 years who is a GHC member, was found to have inherited the *RYR1* pathogenic variant. This patient has never had a surgical procedure.

### Proband 3

The proband was a 63‐year‐old woman found to have a variant in the *LDLR* gene (OMIM 606945), c.1975G>A resulting in p.Val133Ile. Pathogenic variants in *LDLR* are associated with familial hyperlipidemia. Although this variant has been reported in associating with hypercholesterolemia, its allele Exome Aggregation Consortium (ExAC) frequency is 0.0008 (Lek et al. 2016). In silico analysis of two probands with this variant were predicted to be pathogenic (Tichý et al. 2012). Given these findings, this variant was of specific interest, despite being a VUS, as the patient had a history of elevated cholesterol levels, as high as 396 mg/dL. VUS for disorders that the patient suffers from are generally returned. Often co‐segregation in the family is investigated. Her past medical history was remarkable for sleep apnea, chronic renal failure that developed after donating a kidney, osteopenia, and depression.

### Family history 3

The proband's father died at 84 years of age from surgical complications following a repair of peripheral vascular lesion (Fig. 1). The proband's father came from a large family with 10–12 siblings, most of whom were murdered at early ages in the Holocaust. Her mother was living at age 95 and had elevated cholesterol of unknown level, hypertension, glaucoma, and depression. The proband did not feel it was necessary for her mother to have genetic testing given her age and other health issues. The proband had one maternal aunt who died of post‐polio syndrome in her 70's and a maternal uncle with obesity who died of congestive heart failure in his 70's. The maternal grandfather died in his 50's and had coronary heart disease, hypertension, and a stroke The maternal grandmother died in her 80's and had a history of elevated blood sugar levels. The proband had one 68‐year‐old brother who had elevated cholesterol levels treated with statins; this patient did not receive his health care at GHC and his sister did not feel that the genetic finding was significant enough to warrant contacting her brother. Neither the proband nor her brother had biologic children.

## Discussion

In the recent past, genetic testing performed on patients was based on phenotype or a family history, with molecular analysis performed only on pertinent genes. As technology has improved resulting in decreased cost for DNA sequencing, panels of genes, whole genome sequencing, and whole exome sequencing have become more routinely utilized in clinical practice and in research. With the recommendation from the American College of Medical Genetics (ACMG) to disclose additional findings of pathogenic variants identified in genomic sequencing in 56 actionable gene‐disease pairs in clinical care (Green et al. 2013) and similar recommendations for return of actionable findings in research (Jarvik et al. 2014), more probands and their family members will receive genetic test results that were unrelated to the test indication and have medical ramifications.

It is common at health care organizations such as GHC to have multiple family members receive their care within the same system. In the cases described in this report, relatives were contacted by the proband, and in most cases, the individuals at risk proceeded with the indicated genetic testing and follow‐up evaluations. None of the three families experienced significantly disrupted family communication such as estrangement or had minor children who would be at risk for the identified pathogenic variant within their nuclear family. While the proband in family 1 and 2 reached out to their children, it was interesting that neither proband contacted their siblings who have equal genetic risk to their children, nor to more extended family members. The proband in family 3 perhaps correctly did not perceive her results as significant and did not communicate her genetic test results to her mother or brother. This pattern of communication is clearly different than in *BRCA1* and *BRCA2* families where a proband found to have a pathogenic variant in either the *BRCA1* or *BRCA2* gene has a high likelihood to inform her siblings, (71% brothers and 86% sisters) at a rate comparable to informing her children (85% for children 14–18) (Patenaude et al. 2006).

Determining the impact of health care utilization as the result of genetic testing is critical, and will be ongoing for individuals and their family members. For Family 1, *SCN5A* pathogenic variants have been associated with both Brugada syndrome and possibly an increased risk of epilepsy. For the grandson of the proband, the identification of the *SCN5A* pathogenic variant will require ongoing monitoring by cardiology, but may have been lifesaving. While it is uncertain whether this patient's seizures are the result of a cardiac arrhythmia or a reaction to medications given for ADHD and depression, he will require careful consideration and monitoring if a new medication, particularly an antidepressant is required.

Family 2 benefitted from entering pertinent information regarding the *RYR1* pathogenic variant in the EHR for anesthetic and medication precautions and counseling that, includes a recognized increased risk of exertional hyperthermia particularly in warm climates. Members of both Family 1 and 2 with pathogenic variants were encouraged to carry the appropriate medical alert information. The proband of Family 3 was already treated with a statin medication with normalized cholesterol levels and required no additional intervention.

For Family 1 and 2, the identification of the pathogenic variant in the proband or family members revealed an unexpected expanded spectrum of phenotypic findings. *SCN5A* pathogenic variants have been primarily associated with cardiac disease including Brugada syndrome, familial dilated cardiomyopathy, progressive familial heart block, Romano–Ward syndrome, and sick sinus syndrome. The association of *SCN5A* pathogenic variants and epilepsy is not well recognized, but may account for the clinical presentation and findings in the proband's grandson. There is previous published report describing a family where individuals with a *SCN5A* pathogenic variant have both Brugada syndrome and epilepsy (Parisi et al. 2013). In a series of 68 patients with epilepsy who were in good health but died unexpectedly of unknown cause (Sudden Unexpected Death in Epilepsy or SUDEP), postmortem DNA testing identified four individuals with nonsynonymous *SCN5A* variants previously identified to cause long QT‐syndrome (LQTS) in other individuals (Tu et al. 2011).

Pathogenic variants in *RYR1* are known to be associated with autosomal dominant, incomplete penetrance malignant hyperthermia as well as a number of muscle diseases such as centronuclear myopathy, central core disease, and multiminicore disease (Brislin and Theroux 2013). What is less well recognized is the risk of exertional hyperthermia and possible heat stroke in individuals who have *RYR1* pathogenic variants. In the study by Roux‐Buisson et al. (2016), of the 23 individuals in a military cohort who had well‐documented episodes of exertional heat stroke, three of them (13%) had a pathogenic *RYR1* variant. Therefore, identifying individuals who carry a pathogenic *RYR1* variant could have implications for their enrollment in the military service, participation in strenuous activities, as well as avoiding circumstances in daily life that can lead to hyperthermia. The extension of phenotypic spectrum of disease related to genetic alterations will be one of the ongoing discoveries and challenges presented by next‐generation sequencing projects.

For Families 1 and 2, there was marked effort to contact individuals in the immediate family, particularly children and grandchildren. Despite offers of assistance from the medical geneticist and genetic counselors to provide additional copies of genetic test results, compose letters specific to the family's specific genetic finding, and identify the location of nearby genetic clinics, we did not find that the probands contacted siblings or more distant relatives. The barriers for communicating genetic test results were not obvious, as all three probands had contact with their siblings and more extended family members. Motivators for family communication include both a sense of obligation to inform relatives and support seeking behavior (Greenberg and Smith 2016).

The need for support can be correlated with disease severity. For individuals with *BRCA1* and *BRCA2* pathogenic variants, there is a significant risk for breast and ovarian cancer which correlates to the high frequency of contacting family members at genetic risk, both siblings and children. The difference in behavior of our probands who only contacted children and adult grandchildren about their genetic results may be the outcome of the lesser perceived risk associated with *SCN5A, RYR1*, and *LDLR* pathogenic variants. As our sample expands with patients enrolled in eMERGEIII, we will have a larger population to evaluate these and other factors that influence the development of the family network.

Complex legal, ethical, and social issues will arise as more patients receive results from WGS and pathogenic variants are identified. GHC is both the insurance company and health care provider for its members. If a member who has a pathogenic variant in an actionable gene refuses to a contact a family member at a risk, does GHC have a duty to warn the family member and offer the indicated genetic counseling and testing? Conversely, would informing a family member be considered a breach of Health Information and Protected Privacy Act and an individual's rights to privacy and confidentiality? “Trickle‐down” genetic testing and will require thoughtful evaluation by the medical, legal, and ethics community.

For the individual who did not receive their healthcare through GHC, it was extremely time consuming to coordinate testing by their primary care provider. As mention, the son of proband 2 did not want to be seen at by another genetics provider because of an extended wait period for an appointment and the additional cost of the specialty care. It would have been more cost‐effective and time efficient to have the testing ordered through GHC. Collaborative agreements between health care providers and insurance companies would be helpful to mitigate similar situations and streamline health care.

The best practice for contacting family members who are at risk for a highly penetrant genetic conditions is evolving. In the third phase of eMERGE, 2500 additional GHC members who are enrolled participants will be sequenced for over 100 genes, including those 56 deemed actionable by ACMG. Those with a pathogenic or likely pathogenic variant in an actionable gene will be seen by Genetic Services for return of results, a complete family history and identification of other family members at risk. Building on clinical experience of directed genetic testing based on phenotype and experience with research projects performing high‐throughput sequencing, we will broaden our experience in family communication and the health care response to genetic disease.

## Conflict of Interest

The authors report no conflict of interest.

## Funding

The eMERGE Network was initiated and funded by NHGRI through the following grants: U01HG006375 and UO1HG008657 (Group Health Cooperative/University of Washington); U01HG006828 (Cincinnati Children's Hospital Medical Center/Boston Children's Hospital); U01HG006830 (Children's Hospital of Philadelphia); U01HG006389 (Essentia Institute of Rural Health, Marshfield Clinic Research Foundation and Pennsylvania State University); U01HG006382 (Geisinger Clinic); U01HG006379 (Mayo Clinic); U01HG006380 (Icahn School of Medicine at Mount Sinai); U01HG006388 (Northwestern University); U01HG006378 (Vanderbilt University Medical Center); and U01HG006385 (Vanderbilt University Medical Center serving as the Coordinating Center). Additional support came from the State of Washington Life Sciences Discovery fund award to the Northwest Institute of Genetic Medicine and the University of Washington Clinical Sequencing Exploratory Research Program (1U01HG006507 and 5U01HG007307).
